# User experience in the metaverse virtual environment at an academic-scientific nursing event in Brazil

**DOI:** 10.1590/0034-7167-2024-0553

**Published:** 2025-12-08

**Authors:** Sandra Helena Cardoso, Rafael Rodrigo da Silva Pimentel, Crislaine Loqueti Santos Rainho Prado, Raquel Acciarito Motta, Camila Mendes da Silva Souza, Raíssa Ottes Vasconcelos, Marcelo Jose dos Santos, Heloisa Helena Ciqueto Peres

**Affiliations:** I Universidade de São Paulo. São Paulo, São Paulo, Brazil; II Hospital Israelita Albert Einstein, Centro de Estudos, Pesquisa e Prática em APS e Redes. São Paulo, São Paulo, Brazil

**Keywords:** Nursing, Virtual Reality, Congresses as Topic, Brazil, Avatar., Enfermería, Realidad Virtual, Eventos Científicos y de Divulgación, Brasil, Avatar.

## Abstract

**Objectives:**

to analyze user experience in the metaverse during an academic-scientific nursing event in Brazil.

**Methods:**

a quantitative-qualitative documentary study, based on records of a survey applied during an academic-scientific event broadcast in the metaverse. The AttrakDiff questionnaire assessed user experience. Descriptive and inferential analyses were performed considering p<0.05. Qualitative data were categorized using content analysis. The study complied with research ethics standards.

**Results:**

positive experiences were observed in the dimensions assessed. Being in the age group of over 60 years was associated with the pragmatic quality dimension (p=0.012), which had a positive average. The comments had very similar impressions of potentialities and limitations, regardless of prior knowledge of the platform.

**Conclusions:**

the study contributed to understanding user experience in immersive environments applied to nursing, evidencing favorable assessments regarding the metaverse usability and functionality, highlighting its effectiveness among older users.

## RESUMEN

**Objetivos:**

analizar la experiencia del usuario en el metaverso durante un evento académico-científico de enfermería en Brasil.

**Métodos:**

estudio documental cuantitativo-cualitativo, basado en los registros de una encuesta aplicada durante un evento académico-científico transmitido en el metaverso. El cuestionario *AttrakDiff* evaluó la experiencia del usuario. Se realizaron análisis descriptivos e inferenciales considerando un valor de p<0,05. Los datos cualitativos se categorizaron mediante análisis de contenido. El estudio cumplió con las normas éticas de investigación.

**Resultados:**

se observaron experiencias positivas en las dimensiones evaluadas. Ser mayor de 60 años se asoció con la dimensión de calidad pragmática (p=0,012), que presentó un promedio positivo. Los comentarios mostraron impresiones muy similares sobre las potencialidades y limitaciones, independientemente del conocimiento previo de la plataforma.

**Conclusiones:**

el estudio contribuyó a la comprensión de la experiencia del usuario en entornos inmersivos aplicados a enfermería, evidenciando evaluaciones favorables en cuanto a la usabilidad y funcionalidad del metaverso, destacando su efectividad entre usuarios mayores.

## INTRODUCTION

The metaverse refers to a three-dimensional immersive reality that simulates virtual environments, allowing us to transcend the limitations of time and space in the virtual world^(1)^. The term “metaverse” was coined by Neal Stephenson in 1992, and since then, it has shown the potential to revolutionize some sectors such as health and education^(1-3)^. Studies indicate an increase in interest in the subject^(4,5)^.

The literature classifies the metaverse into four types: (1) Augmented reality, an environment based on physical reality that expands the real world where an individual is located, built through intelligent location technologies, such as the Global Positioning System; (2) Lifelogging, technology that allows individuals to capture, store, and share life experiences and daily information about other people or objects, such as Facebook^®^ and Instagram^®^; (3) Mirror worlds, virtual model that reflects the outside world, with the aim of making the real world more efficient and convenient, such as Google Maps^®^ and Zoom^®^; (4) Virtual worlds, virtual world built in a digital environment where individuals can interact through avatars and communicate, such as the online games Roblox^®^ and Minecraft^®^, with the use of glasses or a helmet to enter the virtual world^(5,6)^.

There are three fundamental characteristics for a virtual environment to be classified as a metaverse: interactivity, in which users must be able to communicate in different ways; persistence, in which the metaverse must be a continuous environment, developing and functioning, and even when users leave and reconnect, they can resume their previous activities and conversations; and corporeality, in which users move through the metaverse through an avatar^(7)^.

The metaverse has been recognized as a promising tool for improving healthcare quality and in scientific and educational initiatives, such as in prevention, treatment, education, training and research settings^(8)^, with the potential to add value and new possibilities to the teaching-learning process^(9)^.

In the context of education, a comprehensive systematic review on the use of the metaverse in nursing education identified increased knowledge, self-confidence, engagement, satisfaction and performance in students, thus demonstrating the metaverse’s viability and pedagogical effectiveness for teaching^(10)^. In the exercise of nursing, it is imperative that nurses are prepared to assimilate technological innovations to solve problems, considering the advancement of digital health^(11)^.

In this context, the metaverse’s immersive realism is a fundamental aspect to be considered^(12)^. Therefore, understanding user experience (UX) during the use of the platform is crucial to ensure the success of any immersive system. The virtual environment needs to promote a positive subjective experience for users, beyond conventional usability, encompassing the sensations and emotions evoked by the system. UX is a growing field that uses several approaches, with scales being the most common methods for measuring specific dimensions of UX^(13)^.

Thus, a virtual reality in the metaverse was developed to support the transmission of an academic-scientific event of a graduate program in nursing management in Brazil. This initiative demonstrates an innovative stance aligned with the latest technological trends, with the aim of exploring the metaverse’s potential and limitations as an immersion tool.

Considering the increase in scientific events held online, especially after the pandemic^(14)^, it is believed that this experience attracted participants interested not only in scientific discussions, but also in the application of emerging technologies in nursing. The introduction of this technology in a scientific event aimed to bring nursing close to the metaverse, since in Brazil this is still little explored.

Academic-scientific events are recognized as relevant settings for scientific communication and knowledge transmission among students, professionals and specialists^(14)^. The environment developed in the metaverse for the event offered advanced interactive features, such as virtual exhibition halls, games, videos, networking spaces and discussion rooms. These features created a rich and engaging experience, where participants could interact with their avatar and communicate dynamically. Thus, the platform proved to be modern and effective for the exchange of knowledge and experiences in nursing management, promoting collaboration and innovation.

## OBJECTIVES

To analyze UX in the metaverse during an academic-scientific nursing event in Brazil.

## METHODS

### Ethical aspects

The study was conducted in accordance with national and international ethics guidelines, approved by the Research Ethics Committee of the *Universidade de São Paulo* School of Nursing, and the opinion is attached to this submission. The Informed Consent Form was waived, since the data came from records stored by the graduate program, which made available only non-sensitive and non-identifiable data for the purpose of conducting the research.

### Study design, period and place

This is a quantitative and qualitative documentary study, based on records of a survey applied during an academic-scientific nursing event. The COnsolidated criteria for REporting Qualitative research^(15)^ and STrengthening the Reporting of OBservational studies in Epidemiology^(16)^ were used to guide the methodology of this study.

**Figure 1:**
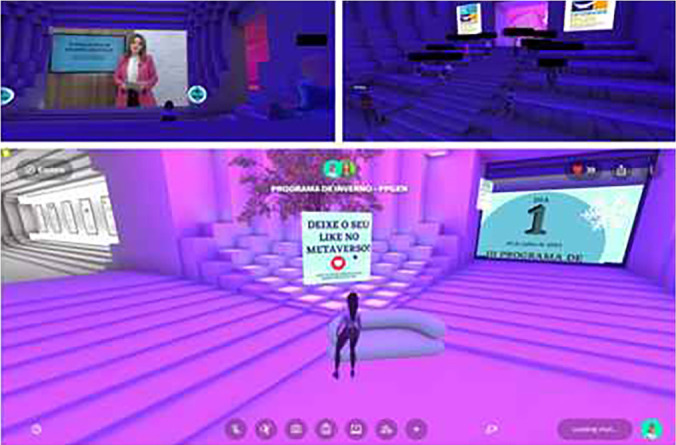
Metaverse Overview of the III Winter Program, São Paulo, São Paulo, Brazil, 2025

A scientific academic event entitled “*III Programa de Inverno do Programa de Pós-Graduação em Gerenciamento em Enfermagem (PPGEn*)”, of the School of Nursing, *Universidade de São Paulo*, was held virtually in July 2023, broadcast on YouTube^®^ and retransmitted to the metaverse, as shown in Figure 1. Participants were able to access the metaverse for the first time on July 6, 2023. Access has remained permanent since this date.

The metaverse, in its free version, was configured by the academic-scientific event organizing committee, which made available recordings of previous editions of the event, an interactive panel so that participants’ avatars could include published articles, games and digital manuals on the event’s theme. Furthermore, participants could watch the event in the metaverse’s own auditorium.

A pre-event was held, with a talk by an expert on the use of the metaverse in nursing and healthcare, and a practical workshop teaching participants how to use the space and interact with the activities on display. No metrics were previously defined regarding the level of interaction of participants and time spent or performing these activities.

### Population, sample; inclusion and exclusion criteria

Participants included undergraduate and graduate students, faculty, nursing professionals, and professionals from other fields of knowledge. They were invited to assess the event platform in the metaverse. Inclusion criteria were being over 18 years of age, participating as an attendee at the event, and completing the assessment form in full.

### Study protocol

Data collection was carried out using Google Forms^®^. The questionnaire was made available in the metaverse environment, in discussion chats, and invitations to complete the questionnaire were sent via email to participants during the event period.

The questionnaire, prepared by the authors, included questions about age range, sex and prior knowledge of the metaverse. Open-ended questions were also reserved to better understand the use of the platform, such as: what were the main potentialities for using technology in the metaverse? What were the main limitations for using metaverse technology?

To assess UX in the metaverse, the AttrakDiff scale was also applied to the questionnaire. It was originally developed in German and later transcribed into English by the authors. It was chosen for its wide use in the UX area, speed and ease of application, in addition to measuring system quality and usability based on UX. This semantic scale contains 28 pairs of opposing adjectives with seven points (Likert scale), in which users choose the point that best represents their experience on the platform^(17,18)^. Authorization for using the scale was obtained from the original authors, who made available the expanded use of their own software to carry out the analyses. The scale has not yet been validated and adapted to the Brazilian context. For the present study, the translation into Portuguese was used according to Rocha^(19)^, and as a complementary measure, the addition of open-ended questions was proposed, aiming to broaden the understanding of participants’ perspective, in order to overcome this limitation.

The scale ranges from -3 (very negative assessment) to +3 (very positive assessment) and 0 (neutral point). Adjectives are distributed across four UX dimensions: pragmatic quality (PQ), which addresses system usability and functionality to capture whether users are manipulating the environment effectively and efficiently; hedonic quality - identification (HQ-I), which quantifies the social interactions between users in the system, whether they are able to express themselves and be perceived by other users within the platform; hedonic quality - stimulation (HQ-S), which assesses psychological well-being, the excitement that the platform provides to users; and attractiveness (ATT), which shows a general analysis that users have of the system and how this affects their judgment as a whole^(17-19)^.

### Analysis of Results and Statistics

Data were analyzed using the statistical software R (version 3.6.1). Qualitative variables were presented as absolute (N) and relative (%) frequencies, while quantitative variables were described by mean, median, and standard deviation. For the Attrakdiff data, item valence standardization was performed to prevent acquiescence during the analysis, and user responses were entered into the Attrakdiff platform (https://www.attrakdiff.de/index_en.html) for graphical representation.

The internal consistency of the Attrakdiff was analyzed, and McDonald’s Omega coefficients were determined, with coefficient values above 0.70 considered acceptable^(20)^.

In the bivariate analysis, nonparametric Mann-Whitney and Kruskal-Wallis statistical tests were applied to examine the association between sociodemographic variables, prior knowledge, and the mean scores of the Attrakdiff dimensions. Statistical significance was considered for p<0.05.

Qualitative data were categorized using Bardin’s content analysis, comprising the following stages: (1) pre-analysis; (2) material exploration and treatment of results; and (3) inferences and interpretation^(21)^.

## RESULTS

In total, 122 users responded to the questionnaire assessing their use of the metaverse. Participants were predominantly female (81.15%), aged between 21 and 40 years (59.84%), and had prior knowledge of accessing the metaverse (62.30%).

**Figure 2:**
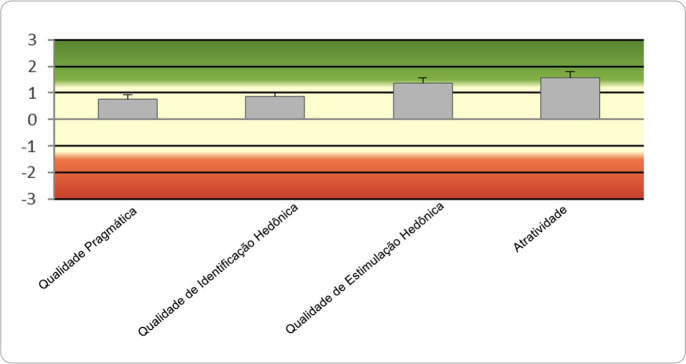
Diagram of the average values of each dimension AttrakDiff, São Paulo, São Paulo, Brazil, 2025

The diagram in Figure 2 illustrates the AttrakDiff questionnaire results with the means of the dimensions assessed. The ATT dimension obtained the highest mean, with 1.57 (SD: 1.30), followed by HQ-S, with 1.37 (SD: 1.16), HQ-I, with 0.85 (SD: 0.92), and PQ, with 0.76 (SD: 1.00).

**Figure 3:**
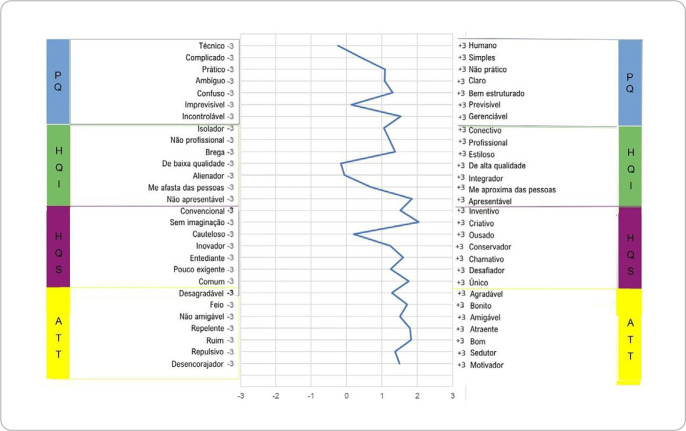
Graphs resulting from AttrakDiff user responses, São Paulo, São Paulo, Brazil, 2025

Figure 3 shows that responses in descriptions by pairs of adjectives presented a distribution to the right (positive), without reaching the extremes (+3 or -3). The results indicated that all dimensions are between 0 and 2 (SD 0.92 and 1.30). Among the pairs of adjectives, the extremes stand out: presentable and creative; technical and low quality.

*PQ - pragmatic quality; HQ-I - hedonic quality - identification; HQ-S - hedonic quality - stimulation; ATT - attractiveness.*

Inferential analysis demonstrated that the age group over 60 years was associated with the PQ dimension (p=0.012). The other variables by dimensions were not statistically significant (Table 1).

**Table 1:** Mean scores of AttrakDiff dimensions by demographic and knowledge factor, São Paulo, São Paulo, Brazil, 2025

Variáveis	Pragmatic quality (PQ)			Hedonic quality-identification (HQ-I)			Hedonic quality- stimulation (HQ-S)			Attractiveness (ATT)		
	M	SD	p^*^	M	SD	p^*^	M	SD	p^*^	M	SD	p^*^
Sex												
Female	0.77	0.96	0.759^a^	0.85	0.91	0.988^a^	1.46	1.11	0.105^c^	1.63	1.24	0.570^c^
Male	0.70	1.18		0.85	0.98		0.98	1.28		1.33	1.55	
Age group												
Up to 20 years	0.74	0.37	0.012^b^	0.83	0.39	0.432^b^	1.07	0.89	0.800^d^	1.93	1.38	
From 21 and 40 years	0.73	1.06		0.93	0.96		1.31	1.26		1.53	1.40	0.890^d^
From 41 and 60 years	0.74	0.97		0.68	0.91		1.51	1.04		1.56	1.16	
More than 60 years	1.76	0.36		1.33	0.46		1.62	0.54		1.90	0.59	
Previous knowledge												
Yes	0.75	1.07	0.964^a^	0.97	1.02	0.298^a^	1.38	1.17	0.909^c^	1.61	1.42	0.587^c^
No	0.76	0.97		0.79	0.86		1.37	1.16		1.55	1.24	

M - mean; SD - standard deviation;

* p=<0.05; a - Student’s t; b- One-way ANOVA (Welch correction); c- Wilcoxon and Mann-Whitney tests; d - Kruskal-Wallis test.

Chart 1 presents comments from users who have accessed the metaverse, which reveal very similar perceptions of potential and limitations, regardless of prior knowledge about the platform.

**Chart 1:** Summary of comments from users who accessed the metaverse, São Paulo, São Paulo, Brazil, 2025

Potential for using technology in the metaverse	Potential for using technology in the metaverse
**With prior knowledge of the metaverse**	
Access to the metaverse after the end of the event; Possibility of exploring scientific materials on the platform for access by users; Potential platform for use in education, teaching and research in nursing and health, integrating technology and innovation; Possibility of networking and interaction through the avatar; Possibility of greater reception, attention to participants, ease of integration, participation.	Access problems due to the devices’ setting and processing capacity, be it a computer, notebook, tablet or cell phone; Instability in the connection to the internet network.
**No prior knowledge of the metaverse**	
Possibility of accessing other resources in the environment, complementing the lectures; Perception of greater proximity and possibility of networking with users; Innovation, pleasant visualization, dynamism and with several possibilities of use and customization; Ease of access even without many skills; Possibility of use in teaching to encourage interactions through avatars; Possibility of use for different areas of research.	Difficulty learning how to use the platform and avatar; Access problems due to the devices’ setting and processing capacity, be it a computer, notebook, tablet or cell phone.

## DISCUSSION

This study, the first of its kind in Brazilian nursing, investigated UX of using the metaverse in an academic-scientific event. The results demonstrated positive experiences, with emphasis on two dimensions (ATT and HQ-S), and indicated that participants considered access to the event to be attractive and with pleasant and satisfactory social experiences. User satisfaction is a crucial predictor in the adoption and maintenance of the use of emerging technologies^(22)^.

Qualitative analysis also revealed positive user perceptions about the potential and limitations of using the metaverse. The literature supports our findings, and in nursing, positive experiences using the metaverse ensured greater self-efficacy in nurses’ work and student learning by providing pleasurable perceptions^(23)^.

In this study, the association of the age group over 60 years with significantly higher attribution in the PQ dimension stands out. Supporting our findings, studies highlight the metaverse’s potential to promote well-being and engagement among older people, with significant cognitive, affective and social benefits, through digital immersion and interaction^(24)^.

UX is multifaceted and can be influenced by factors such as usability and emotional aspects, considering the experience holistically beyond functionality^(18)^. Interactions in the virtual environment shape the experience and engagement of participants^(23)^.

It was observed that few pairs of AttrakDiff adjectives differed from the others in the assessments, notably in the PQ and HQ-S dimensions. The pairs “technical - human” and “cautious - bold” presented averages closer to neutral, indicating a possible semantic difficulty in the assessment of adjectives for the context of an academic event in the metaverse, a result similar to that described in literature^(25)^. In view of this, the importance of reviewing and semantically adjusting the adjective pairs and culturally adapting the instrument to be used is reinforced, considering the target audience and the nature of the experience analyzed.

The PQ dimension, despite being positive, presented the lowest average among the dimensions assessed, which indicates the perception of limitations in the ease of use of the platform. This result suggests that, despite the metaverse’s innovative and immersive nature, usability emerges as a factor to be improved in UX. The literature describes challenges related to the learning curve and the complexity of navigation in emerging technologies^(26)^. Therefore, it is essential to consider user-centered design principles when building these environments, to optimize usability and the positive impact of the immersive experience.

Thus, according to users’ perceptions, the metaverse has limitations, such as poor platform handling skills coupled with instability of access, due to the type of device and internet connectivity. The literature confirms that the type of device can interfere with UX, and to enhance immersion, good equipment and a high-speed network are necessary^(26,27)^.

Users’ impressions of potential and limitations were very similar, regardless of whether they had prior knowledge of the platform or not. Among the potentialities, innovation and interaction with both the materials provided and with other users stand out. Innovation is a latent perception, as the metaverse offers individuals more opportunities to experiment, explore, learn and teach, in addition to enabling interaction with people and practice in contexts that they would not be able to experience in the real world^(6,22)^.

To expand the use of the metaverse in academic-scientific events, it is essential to invest in implementing improvements in connectivity technology, in order to enable, especially in developing countries, the population’s access to the internet and digital technologies^(28)^. The COVID-19 pandemic has accelerated the adoption of emerging technologies in health and education^(2,5,11)^, but it has also highlighted inequalities in digital access. For a more inclusive future, it is essential to reflect on the training of healthcare professionals with skills development for managing emerging technologies and applying strategies for equity in access to digital technologies^(2,5,11)^.

### Study limitations

Among the limitations of this study, it is worth noting that the scale used was not subjected to cultural adaptation for the Brazilian context. In addition, users were not informed about the meanings of the instrument’s items, which may have generated doubts during completion. Despite these limitations, it is worth noting that the scale is widely used by Brazilian UX researchers, especially in computer science^(18)^, and that qualitative aspects contributed to improving participants’ understanding of their potentialities and limitations in using the metaverse.

### Contributions to nursing

In this study, we understand that the use of the metaverse promoted positive experiences for those registered for the academic-scientific event, which is a historic milestone in innovation in Brazilian nursing. This proposal is the beginning of understanding the nursing users’ experience in the metaverse, which can be created for various purposes and applicable in different contexts of the profession. In education, it enables virtual simulations in settings such as operating rooms, Intensive Care Units, care for multiple victims, and actions in communities. In healthcare, the metaverse can be applied in teleconsultations, assessments, and educational actions aimed at health promotion and rehabilitation. Moreover, the use of visual and interactive resources allows for more engaging experiences, such as meditative practices and gamified physical activities, which can positively contribute to users’ quality of life.

## CONCLUSIONS

This study innovates by exploring UX in the nursing metaverse, and reveals predominantly positive assessments. Participants perceived this virtual environment as attractive, socially pleasant and capable of fostering engagement through immersive experiences, which reinforces the potential of this technology as an educational tool in nursing.

It is recommended that future research focus on the AttrakDiff scale cultural adaptation and validity for the Brazilian context, due to its international robustness in assessing UX in virtual environments. In addition, longitudinal studies are needed to deepen the understanding of the determinants of UX in the metaverse in different settings of nursing training and professional practice.

The metaverse’s potential to be incorporated into interactive scientific events stands out, especially in contexts with difficult geographical access. When integrated into the teaching-learning process, the metaverse can contribute significantly to democratization of knowledge in nursing, promotion of digital literacy and strengthening of equity in access to health education in Brazil.

## Data Availability

The research data are available only upon request.
