# Supplementary material for: User experience in the metaverse virtual environment at an academic-scientific nursing event in Brazil
Source: Rev Bras Enferm. 2025 Dec 8;78(5):e20240553. doi: 10.1590/0034-7167-2024-0553 (PMC12707772; doi:10.1590/0034-7167-2024-0553)
Supplement: Translation

# Translation: Experiência de usuários no ambiente virtual do metaverso em evento acadêmico-científico de enfermagem no Brasil

**DOI:** 10.1590/0034-7167-2024-0553pt

**Authors:** Sandra Helena Cardoso, Rafael Rodrigo da Silva Pimentel, Crislaine Loqueti Santos Rainho Prado, Raquel Acciarito Motta, Camila Mendes da Silva Souza, Raíssa Ottes Vasconcelos, Marcelo Jose dos Santos, Heloisa Helena Ciqueto Peres

**Affiliations:** I Universidade de São Paulo. São Paulo, São Paulo, Brasil; II Hospital Israelita Albert Einstein, Centro de Estudos, Pesquisa e Prática em APS e Redes. São Paulo, São Paulo, Brasil

**Keywords:** Enfermagem, Realidade Virtual, Eventos Científicos e de Divulgação, Brasil, Avatar.

## Abstract

**Objetivos:**

analisar a experiência do usuário no metaverso durante evento acadêmico-científico de enfermagem no Brasil.

**Métodos:**

estudo quanti-qualitativo documental, a partir dos registros de uma *survey* aplicada durante evento acadêmico-científico transmitido no metaverso. O questionário *AttrakDiff* avaliou a experiência do usuário. Foram realizadas análises descritivas e inferenciais considerando p<0,05. Os dados qualitativos foram categorizados por meio da análise de conteúdo. O estudo atendeu às normas de ética em pesquisa.

**Resultados:**

observaram-se experiências positivas nas dimensões avaliadas. Estar na faixa etária de mais de 60 anos esteve associada à dimensão qualidade pragmática (p=0,012), que teve uma média positiva. Os comentários tinham impressões de potencialidades e limitações muito similares, independentemente do conhecimento prévio da plataforma.

**Conclusões:**

o estudo contribuiu para compreensão da experiência do usuário em ambientes imersivos aplicados à enfermagem, evidenciando avaliações favoráveis quanto à usabilidade e funcionalidade do metaverso, com destaque para sua efetividade entre usuários idosos.

## INTRODUÇÃO

O metaverso refere-se a uma realidade imersiva tridimensional que simula ambientes virtuais, permitindo transcender as limitações de tempo e espaço no mundo virtual^(1)^. O termo “metaverso” foi cunhado por Neal Stephenson em 1992, e desde então, vem apresentando potencial para revolucionar alguns setores como saúde e educação^(1-3)^. Estudos apontam aumento no interesse pela temática^(4,5)^.

A literatura classifica o metaverso em quatro tipos: (1) Realidade aumentada, ambiente baseado na realidade física que expande o mundo real onde um indivíduo está localizado, construído por meio de tecnologias inteligentes de localização, como o *Global Positioning System*; (2) Registro de vida, tecnologia que permite que o indivíduo capture, armazene e compartilhe experiências da sua vida e informações diárias sobre outras pessoas ou objetos, como o *Facebook* ^®^ e *Instagram* ^®^; (3) Mundo espelhado, modelo virtual que reflete o mundo exterior, com o intuito de tornar o mundo real mais eficiente e conveniente, como o *Google Maps* ^®^ e *Zoom* ^®^; (4) Realidade virtual, mundo virtual construído em um ambiente digital onde os indivíduos podem interagir por meio de avatares e se comunicar, como os jogos *online Roblox* ^®^ e *Minecraft* ^®^, com o uso de óculos ou capacete para adentrar o mundo virtual^(5,6)^.

Há três características fundantes para que um ambiente virtual seja enquadrado como metaverso: interatividade, em que os usuários devem ser capazes de se comunicar de diferentes formas; persistência, em que o metaverso deve ser um ambiente contínuo, desenvolver-se e funcionar, e mesmo quando os usuários saem e se reconectam, eles conseguem retomar suas atividades e conversas anteriores; e corporeidade, em que os usuários se movimentam pelo metaverso através de um avatar^(7)^.

O metaverso tem sido reconhecido como uma ferramenta promissora para melhoria da qualidade da assistência à saúde e em iniciativas científicas e educacionais, como nos cenários de prevenção, tratamento, educação, treinamento e pesquisa^(8)^, com potencial de agregar valor e novas possibilidades ao processo de ensino-aprendizagem^(9)^.

No âmbito da educação, revisão sistemática abrangente sobre o uso do metaverso no ensino de enfermagem identificou o aumento do conhecimento, autoconfiança, engajamento, satisfação e desempenho em estudantes, evidenciando, assim, a viabilidade e efetividade pedagógica do metaverso para o ensino^(10)^. No exercício da profissão, é imperativo que os enfermeiros estejam preparados para assimilar as inovações tecnológicas para a resolução de problemas, considerando o avanço da saúde digital^(11)^.

Nesse contexto, o realismo imersivo do metaverso é um aspecto fundamental a ser considerado^(12)^. Por isso, compreender a experiência do usuário (*user experience*, UX) durante o uso da plataforma é crucial para garantir o sucesso de qualquer sistema imersivo. O ambiente virtual precisa promover uma experiência subjetiva positiva aos usuários, além da usabilidade convencional, abrangendo as sensações e emoções evocadas pelo sistema. A UX é um campo em expansão que utiliza diversas abordagens, sendo as escalas os métodos mais comuns para medir dimensões específicas da UX^(13)^.

Dessa forma, foi desenvolvida uma realidade virtual no metaverso para apoiar a transmissão de evento acadêmico-científico de um programa de pós-graduação em gerenciamento em enfermagem no Brasil. Essa iniciativa demonstra postura inovadora e alinhada às últimas tendências tecnológicas, com objetivo de explorar as potencialidades e limitações do metaverso enquanto ferramenta de imersão.

Considerando o aumento na realização dos eventos científicos em ambiente *online*, principalmente após a pandemia^(14)^, acredita-se que essa experiência tenha atraído participantes interessados não apenas nas discussões científicas, mas também na aplicação de tecnologias emergentes na enfermagem. A introdução dessa tecnologia em evento científico visou promover a aproximação da enfermagem com o metaverso, visto que no Brasil este ainda é pouco explorado.

Os eventos acadêmicos-científicos são reconhecidos como relevantes cenários de comunicação científica, e transmissão de conhecimento entre estudantes, profissionais e especialistas^(14)^. O ambiente desenvolvido no metaverso para o evento ofereceu recursos interativos avançados, como salas de exposições virtuais, jogos, vídeos, espaços de *networking* e salas de discussão. Essas funcionalidades criaram uma experiência rica e envolvente, onde o participante poderia interagir com o seu avatar e se comunicar de forma dinâmica. Assim, a plataforma se mostrou moderna e eficaz para a troca de conhecimentos e experiências na área de gerenciamento em enfermagem, promovendo colaboração e inovação.

## OBJETIVOS

Analisar a UX no metaverso durante a realização de um evento acadêmico-científico de enfermagem no Brasil.

## MÉTODOS

### Aspectos éticos

O estudo foi conduzido de acordo com as diretrizes de ética nacionais e internacionais, aprovado pelo Comitê de Ética em Pesquisa da Escola de Enfermagem da Universidade de São Paulo, parecer anexo à presente submissão. O Termo de Consentimento Livre e Esclarecido foi dispensado, pois os dados são oriundos dos registros armazenados pelo programa de pós-graduação, que disponibilizou apenas os dados não sensíveis e não identificáveis para a realização da pesquisa.

### Desenho, período e local do estudo

Estudo quanti-qualitativo documental, a partir dos registros de uma *survey* aplicada durante evento acadêmico-científico de enfermagem. Foram utilizados os instrumentos *COnsolidated criteria for REporting Qualitative research* ^(15)^ e *STrengthening the Reporting of OBservational studies in Epidemiology* ^(16)^ para nortear a metodologia deste estudo.

O evento acadêmico científico intitulado “III Programa de Inverno do Programa de Pós-Graduação em Gerenciamento em Enfermagem (PPGEn)”, da Escola de Enfermagem da Universidade de São Paulo, foi realizado na modalidade virtual em julho de 2023, com transmissão na plataforma *YouTube* ^®^ e retransmissão para o metaverso, conforme Figura 1. Os participantes puderam acessar pela primeira vez o metaverso no dia 06/07/2023. O acesso se mantém permanente desde esta data.

**Figura 1:** Visão geral do metaverso do III Programa de Inverno, São Paulo, São Paulo, Brasil, 2025

O metaverso, em sua versão gratuita, foi parametrizada pela comissão organizadora do evento acadêmico-científico, que disponibilizou as gravações das edições anteriores do evento, painel interativo para que os avatares dos participantes pudessem incluir artigos publicados, jogos e manuais digitais sobre a temática do evento. Além disso, os participantes poderiam assistir ao evento no próprio auditório do metaverso.

Um pré-evento foi promovido contendo na programação uma palestra com especialista sobre uso do metaverso na enfermagem e saúde, e oficina prática ensinando os participantes a usarem o espaço e interagir com as atividades ali expostas. Não foram previamente delimitadas métricas quanto ao nível de interação dos participantes e ao tempo de permanência ou de realização dessas atividades.

### População, amostra; critérios de inclusão e exclusão

Os participantes incluíram estudantes de graduação e pós-graduação, docentes, profissionais de enfermagem e de outras áreas do conhecimento. Esses foram convidados a avaliar a plataforma do evento no metaverso. Os critérios de inclusão foram ter idade superior a 18 anos, participação como ouvinte no evento e preenchimento completo do formulário de avaliação.

### Protocolo do estudo

A coleta de dados foi realizada por meio do *Google Forms* ^®^. O questionário foi disponibilizado no ambiente do metaverso, nos *chats* de discussão e enviados convites para o preenchimento por e-mail aos participantes durante o período de realização do evento.

O questionário, elaborado pelos autores, incluiu perguntas sobre faixa etária, sexo e conhecimento prévio do metaverso. Ainda foram reservadas questões abertas objetivando maior compreensão do uso da plataforma como: quais foram as principais potencialidades para uso da tecnologia no metaverso? Quais foram as principais limitações para uso da tecnologia do metaverso?

Para avaliar a UX no metaverso, também foi aplicado no questionário a escala *AttrakDiff*, desenvolvida originalmente no idioma alemão e posteriormente transcrita para a língua inglesa pelos autores, eleita por sua ampla utilização na área UX, rapidez e facilidade na aplicação, além de medir qualidade e usabilidade do sistema a partir da UX. Essa escala semântica contém 28 pares de adjetivos opostos com sete pontos (escala Likert), em que o usuário elege o ponto que melhor representa sua experiência na plataforma^(17,18)^. Obteve-se a autorização para o uso da escala pelos autores originais, que disponibilizaram a ampliação do uso do *software* próprio para procedermos às análises. A escala ainda não foi validada e adaptada para o contexto brasileiro. Para o presente estudo, foi utilizada a tradução no idioma português, conforme Rocha^(19)^, e de maneira complementar, propôs-se o acréscimo das questões abertas, visando ampliar a compreensão sobre a perspectiva dos participantes, de modo a transpor esta limitação.

A escala varia de -3 (avaliação muito negativa) a +3 (avaliação muito positiva) e 0 (ponto neutro). Os adjetivos estão distribuídos em quatro dimensões de UX: qualidade pragmática (PQ), que aborda a usabilidade e funcionalidade do sistema para captar se os usuários estão manipulando o ambiente com eficácia e eficiência; qualidade de identificação hedônica (HQ-I), que quantifica as interações sociais entre os usuários no sistema, se estes conseguem se expressar e ser percebidos por outros usuários dentro da plataforma; qualidade de estimulação hedônica (HQ-S), que avalia o bem-estar psicológico, a excitação que a plataforma proporciona ao usuário; e atratividade (ATT), que mostra uma análise geral que o usuário tem do sistema e como isso afeta o julgamento como um todo^(17-19)^.

### Análise dos resultados e estatística

Os dados foram analisados utilizando o software estatístico R (versão 3.6.1). As variáveis qualitativas foram apresentadas como frequências absolutas (N) e relativas (%), enquanto as variáveis quantitativas por meio de média, mediana e desvio padrão. Para os dados do Attrakdiff, foi realizada a padronização da valência dos itens para evitar a aquiescência durante a análise, e as respostas dos usuários foram inseridas na plataforma do Attrakdiff (https://www.attrakdiff.de/index-en.html) para representação gráfica.

A consistência interna do Attrakdiff foi analisada, e os coeficientes alpha de Ômega de McDonald foram determinados, com valores de coeficientes acima de 0,70 como aceitáveis^(20)^.

Na análise bivariada foram aplicados testes estatísticos não paramétricos de Mann-Whitney e Kruskal-Wallis para analisar a associação entre as variáveis sociodemográficas, conhecimento prévio e a média das dimensões do Attrakdiff. A significância estatística foi considerada para p<0,05.

Os dados qualitativos foram categorizados por meio da análise de conteúdo de Bardin, compreendida nas etapas: 1) pré-análise; 2) exploração do material e tratamento dos resultados; 3) inferências e interpretação^(21)^.

## RESULTADOS

No total, 122 usuários responderam ao questionário avaliando o uso do metaverso. Os participantes eram predominantemente do sexo feminino (81,15%), com faixa etária de 21 a 40 anos (59,84%), e tinham conhecimento prévio de acesso ao metaverso (62,30%).

O diagrama da Figura 2 ilustra os resultados do questionário *AttrakDiff* com as médias das dimensões avaliadas. A dimensão ATT obteve a maior média, com 1,57 (DP: 1,30), seguida por HQ-S, com 1,37 (DP: 1,16), HQ-I, com 0,85 (DP: 0,92), e PQ, com 0,76 (DP: 1,00).

**Figura 2:** Diagrama dos valores média de cada dimensão *AttrakDiff*, São Paulo, São Paulo, Brasil, 2025

Na Figura 3, observa-se que as respostas nas descrições por pares de adjetivos apresentaram uma distribuição para direita (positiva), sem alcançar os extremos (+3 ou -3). Os resultados indicaram que todas as dimensões estão entre 0 e 2 (DP 0,92 e 1,30). Entre os pares de adjetivos, destacam-se os extremos: apresentável e criativo; técnico e de baixa qualidade.

**Figura 3:** Gráficos resultantes das respostas dos usuários*AttrakDiff*, São Paulo, São Paulo, Brasil, 2025

*PQ - qualidade pragmática; HQ-I - qualidade de identificação hedônica; HQ-S - qualidade de estimulação hedônica; ATT - atratividade.*

A análise inferencial demonstrou que a faixa etária mais de 60 anos esteve associada à dimensão PQ (p=0,012). As demais variáveis por dimensões não foram significativas estatisticamente (Tabela 1).

**Tabela 1:** Pontuações médias das dimensões do *AttrakDiff* por fator demográfico e conhecimento, São Paulo, São Paulo, Brasil, 2025

Variáveis	Qualidade pragmática (PQ)			Qualidade de identificação hedônica (HQ-I)			Qualidade de estimulação hedônica (HQ-S)			Atratividade (ATT)		
	M	DP	p^*^	M	DP	p^*^	M	DP	p^*^	M	DP	p^*^
Sexo												
Feminino	0,77	0,96	0,759^a^	0,85	0,91	0,988^a^	1,46	1,11	0,105^c^	1,63	1,24	0,570^c^
Masculino	0,70	1,18		0,85	0,98		0,98	1,28		1,33	1,55	
Faixa etária												
Até 20 anos	0,74	0,37	0,012^b^	0,83	0,39	0,432^b^	1,07	0,89	0,800^d^	1,93	1,38	
De 21 e 40 anos	0,73	1,06		0,93	0,96		1,31	1,26		1,53	1,40	0,890^d^
De 41 e 60 anos	0,74	0,97		0,68	0,91		1,51	1,04		1,56	1,16	
Mais de 60 anos	1,76	0,36		1,33	0,46		1,62	0,54		1,90	0,59	
Conhecimento prévio												
Sim	0,75	1,07	0,964^a^	0,97	1,02	0,298^a^	1,38	1,17	0,909^c^	1,61	1,42	0,587^c^
Não	0,76	0,97		0,79	0,86		1,37	1,16		1,55	1,24	

M - média; DP - desvio padrão;

* p=<0,05; a - t de Student; b- One-way ANOVA (correção de Welch); ctestes de Wilcoxon e Mann-Whitney; d - teste de Kruskal-Wallis.

O Quadro 1 apresenta os comentários dos usuários que acessaram o metaverso, os quais revelam percepções de potencialidades e limitações muito similares, independentemente do conhecimento prévio sobre a plataforma.

**Quadro 1:** Síntese dos comentários dos usuários que acessaram o metaverso, São Paulo, São Paulo, Brasil, 2025

Potencialidades para uso da tecnologia no metaverso	Limitações para uso da tecnologia no metaverso
**Com conhecimento prévio do metaverso**	
Acesso ao metaverso após o término do evento; Possibilidade de explorar materiais científicos na plataforma para acesso pelos usuários; Plataforma potencial para uso na educação, ensino e pesquisa na enfermagem e saúde, integrando tecnologia e inovação; Possibilidade de *networking* e interação por meio do avatar; Possibilidade de maior acolhimento, atenção aos participantes, facilidade de integração, participação.	Problemas de acesso devido à configuração e capacidade de processamento dos dispositivos, seja computador, *notebook, tablet* ou celular; Instabilidade na conexão com a rede de internet.
**Sem conhecimento prévio do metaverso**	
Possibilidade de acesso a outros recursos no ambiente, complementando as palestras; Percepção de maior proximidade e possibilidade de *networking* com usuários; Inovação, visualização agradável, dinamismo e com diversas possibilidades de uso e customização; Facilidade de acesso e mesmo sem muitas habilidades; Possibilidade de uso no ensino para incentivar interações por meio dos avatares; Possibilidade de uso para diferentes áreas de pesquisa.	Dificuldade para aprender a manejar a plataforma e o avatar; Problemas de acesso devido à configuração e capacidade de processamento dos dispositivos, seja computador, *notebook, tablet* ou celular.

## DISCUSSÃO

Este estudo, inédito na enfermagem brasileira, investigou a UX com o uso do metaverso em evento acadêmico-científico. Os resultados demonstraram experiências positivas, com destaque para duas dimensões (ATT e HQ-S), e aponta que os participantes consideraram o acesso ao evento de maneira atrativa e com experiências sociais agradáveis e satisfatórias. A satisfação do usuário é um preditor crucial na adoção e manutenção do uso de tecnologias emergentes^(22)^.

A análise qualitativa também revelou percepções positivas dos usuários sobre as potencialidades e limitações no uso do metaverso. A literatura corrobora nossos achados, sendo que, na enfermagem, as experiências positivas no uso do metaverso garantiram maior autoeficácia no trabalho dos enfermeiros e aprendizado de estudantes, por proporcionar percepções prazerosas^(23)^.

No presente estudo, destaca-se a associação da faixa etária acima de 60 anos com a atribuição significativamente maior na dimensão PQ. Corroborando nossos achados, estudos evidenciam o potencial do metaverso para promover bem-estar e engajamento entre pessoas idosas, com benefícios cognitivos, afetivos e sociais significativos, por meio da imersão e interação digital^(24)^.

A UX é multifacetada e pode ser influenciada por fatores como a usabilidade e aspectos emocionais, considerando a experiência de forma holística para além da funcionalidade^(18)^. As interações no ambiente virtual moldam a experiência e o envolvimento dos participantes^(23)^.

Observou-se que poucos pares de adjetivos do *AttrakDiff* se diferenciam dos demais nas avaliações, notadamente nas dimensões PQ e HQ-S. Os pares “técnico - humano” e “cauteloso - ousado” apresentaram médias mais próximas do neutro, apontando possível dificuldade semântica na avaliação dos adjetivos para o contexto de um evento acadêmico no metaverso, resultado semelhante ao descrito na literatura^(25)^. Diante disso, reforça-se a importância de revisar e ajustar semanticamente os pares de adjetivos e adaptar culturalmente o instrumento a ser utilizado, considerando o público-alvo e a natureza da experiência analisada.

A dimensão PQ, apesar de positiva, apresentou a menor média entre as dimensões avaliadas, o que indica a percepção de limitações na facilidade de uso da plataforma. Esse resultado sugere que, apesar do caráter inovador e imersivo do metaverso, a usabilidade desponta como fator a ser aprimorado na UX. A literatura descreve desafios relacionados à curva de aprendizagem e à complexidade de navegação em tecnologias emergentes^(26)^. Assim, torna-se essencial considerar princípios de *design* centrado no usuário na construção desses ambientes, para otimizar a usabilidade e o impacto positivo da experiência imersiva.

Dessa forma, segundo as percepções dos usuários, o metaverso apresenta limitações, como pouca destreza de manejo da plataforma atrelada à instabilidade de acesso, devido ao tipo de dispositivo e à conectividade da internet. A literatura confirma que o tipo de dispositivo pode interferir na UX, e para potencializar a imersão, é necessário um bom equipamento e rede de alta velocidade^(26,27)^.

As impressões de potencialidades e limitações dos usuários foram muito similares, independentemente de ter conhecimento prévio ou não da plataforma. Entre as potencialidades, destacam-se inovação e interação tanto com os materiais disponibilizados quanto com outros usuários. A inovação é uma percepção latente, pois o metaverso oferece aos indivíduos mais oportunidades de experimentar, explorar, aprender e ensinar, além de possibilitar a interação com as pessoas e praticar em contextos que não poderiam experimentar no mundo real^(6,22)^.

Para ampliar o uso do metaverso em eventos acadêmicos-científicos, é fundamental investir na implementação de melhorias na tecnologia de conectividade, de modo a viabilizar, especialmente em países em desenvolvimento, o acesso da população à internet e às tecnologias digitais^(28)^. A pandemia de COVID-19 acelerou a adoção de tecnologias emergentes na saúde e educação^(2,5,11)^, mas também evidenciou as desigualdades no acesso digital. Para um futuro mais inclusivo, é essencial refletir sobre a formação dos profissionais de saúde com desenvolvimento de competências para manejo das tecnologias emergentes e aplicação de estratégias para a equidade no acesso às tecnologias digitais^(2,5,11)^.

### Limitações do estudo

Entre as limitações deste estudo, destaca-se que a escala utilizada não foi submetida à adaptação cultural para o contexto brasileiro. Além disso, os usuários não foram esclarecidos quanto aos significados dos itens do instrumento, o que pode ter gerado dúvidas durante o preenchimento. Apesar dessas limitações, ressalta-se que a escala é amplamente utilizada pelos pesquisadores brasileiros de UX, principalmente na área das ciências da computação^(18)^, e que os aspectos qualitativos contribuíram para melhorar a compreensão dos participantes sobre suas potencialidades e limitações no uso do metaverso.

### Contribuições para a área da enfermagem

Compreendemos neste estudo que o uso do metaverso promoveu experiências positivas dos inscritos no evento acadêmico-científico, o que é um marco histórico de inovação que nasce na enfermagem brasileira. Essa proposta é o início para compreender a experiência dos usuários da enfermagem no metaverso que podem ser criados para diversas finalidades, com aplicabilidade em diferentes contextos da profissão. Na educação, possibilita simulações virtuais em cenários de salas de cirurgia, Unidades de Terapia Intensiva, atendimentos a múltiplas vítimas e ações em comunidades. Na assistência, o metaverso pode ser aplicado nas teleconsultas, avaliações e ações educativas voltadas à promoção da saúde e à reabilitação. Além disso, a utilização de recursos visuais e interativos permite experiências mais engajadoras, como práticas meditativas, atividades físicas gamificadas, as quais podem contribuir positivamente para a qualidade de vida dos usuários.

## CONCLUSÕES

O presente estudo inova ao explorar a experiência de usuários no metaverso na enfermagem, e revela avaliações predominantemente positivas. Os participantes perceberam esse ambiente virtual como atrativo, socialmente agradável e capaz de favorecer o engajamento por meio de experiências imersivas, o que reforça o potencial dessa tecnologia como ferramenta educacional na área da enfermagem.

Recomenda-se que futuras pesquisas se dediquem à adaptação cultural e validação da escala *AttrakDiff* para o contexto brasileiro, devido à sua robustez internacional na avaliação da UX em ambientes virtuais. Além disso, estudos longitudinais são necessários para aprofundar a compreensão sobre os determinantes da UX no metaverso em diferentes cenários da formação e atuação profissional em enfermagem.

Destaca-se o potencial do metaverso para ser incorporado em eventos científicos interativos, especialmente em contextos de difícil acesso geográfico. Ao ser integrado ao processo de ensino-aprendizagem, o metaverso pode contribuir significativamente para a democratização do conhecimento em enfermagem, a promoção do letramento digital e o fortalecimento da equidade no acesso à formação em saúde no Brasil.

## Data Availability

Os dados de pesquisa só estão disponíveis mediante solicitação.
